# Clinical and Sociodemographic Characteristics of Patients with Peripheral Facial Paralysis in Medical Rehabilitation: A Comprehensive Description

**DOI:** 10.3390/medicina61050925

**Published:** 2025-05-20

**Authors:** María Navarro-Martínez, Paula Rodríguez-Fernández, Sandra Núñez-Rodríguez, Jerónimo Javier González-Bernal

**Affiliations:** 1 Rehabilitation Service of Río Hortega University Hospital, 47012 Valladolid, Spain; mnavarroma@saludcastillayleon.es; 2 Faculty of Legal Sciences, Education, and Humanities, Universidad Europea de Madrid, Villaviciosa de Odón, 28670 Madrid, Spain; 3 Department of Health Sciences, University of Burgos, 09001 Burgos, Spain; snr1005@alu.ubu.es (S.N.-R.); jejavier@ubu.es (J.J.G.-B.)

**Keywords:** peripheral facial paralysis, rehabilitation, facial disability, Sunnybrook Facial Grading System

## Abstract

*Background and Objectives*: Peripheral facial paralysis (PFP) is a condition with diverse etiologies, requiring multidisciplinary management. This study aimed to describe the sociodemographic, clinical, and functional characteristics of patients with PFP treated at the Rehabilitation Service of the University Hospital of Burgos and to evaluate factors associated with the initial degree of impairment. *Materials and Methods*: A descriptive, cross-sectional study was conducted on 45 patients referred to the Rehabilitation Service from July 2018 to July 2023. Inclusion criteria included first-time rehabilitation visits for PFP during the study period with signed informed consent. Patients with prior PFP on the affected side or severe comorbidities, such as stroke, were excluded. Data were collected from medical records and initial evaluations. The Sunnybrook Facial Grading System (SFGS) was used to assess impairment. *Results*: Idiopathic paralysis was the most common etiology, with a predominance in men (60.9%) and middle-aged or older adults. Otorhinolaryngology was the leading referral service, though primary care referrals were underrepresented. Delays in initiating rehabilitation were identified, especially in complex cases like acoustic neurinoma. The ANOVA test revealed no significant differences in functional assessments based on age, sex, or etiology, likely due to the limited sample size. *Conclusions*: The study highlights the predominance of idiopathic etiology in PFP and the importance of otorhinolaryngology in referrals. Greater awareness in primary care and early identification are crucial. Future studies with larger samples are needed to evaluate predictors of impairment and optimize rehabilitation strategies.

## 1. Introduction

Peripheral facial paralysis (PFP) is a relatively common neurological condition in the general population, with an estimated annual incidence of 23 per 100,000 inhabitants. It occurs due to an impairment of the facial nerve, from its emergence from the brainstem at the pontomedullary sulcus to its innervation regions and can be damaged at any point along this path [1]. It involves weakness or loss of function in the muscles innervated by the facial nerve, corresponding to the seventh cranial nerve, which is a mixed nerve composed of 58% motor fibers, 24% parasympathetic fibers, and 18% sensory fibers [2]. This results in a partial or complete loss of voluntary facial mobility, the inability to close the eyelid, and deviation of the mouth corner to the contralateral side [1,2,3].

It is classified as idiopathic (primary), which is the most common type and is associated with herpes simplex virus type I, or secondary, linked to neurological conditions, infections, neoplasms, autoimmune diseases, or trauma [3]. Additionally, it can be classified as unilateral or bilateral based on its extent and as complete or partial depending on the degree of muscle involvement [3].

The main risk factors for the development of PFP are pregnancy, especially during the third trimester or the first week after delivery, upper respiratory tract infections such as the flu or a cold, diabetes mellitus (DM), high blood pressure and obesity, which often coincide with the etiology [1]. It also often includes difficulty closing the eyelids, problems with eating, alterations in facial expression, and the emotional impact of facial disfigurement [1]. Therefore, the main treatment objectives are to correct facial disfigurement, relieve associated pain, and address the functional and psychological disorders caused by the condition [4].

Initially, it is essential to inform the patient about the nature of the disease, its potential evolution, and treatment options, which helps reduce anxiety and promotes adherence to the therapeutic plan [1,5].

Although there is currently no consensus on the correct therapeutic management of PFP, there is agreement on the importance of early initiation of treatment. The main aspects of medical treatment are general measures and specific treatment of the etiology Steroid treatment is the most used [1,5]. The pharmacological management of PFP is carried out in the emergency department, as it is within the first 72 h of the onset of the condition that corticosteroid treatment is administered for 7 to 10 days, and in certain cases, an antiviral is added. The use of any other medication is not described in the guidelines for the management of PFP [1,5].

Additionally, specialized rehabilitation is a key component of treatment [6,7,8]. This begins with an evaluation of facial symmetry, voluntary and involuntary mobility, and the patient’s emotional state. Interventions include physiotherapy, neuromuscular retraining, and facial exercises designed to restore muscle strength and control [9]. Techniques such as electrical stimulation, acupuncture, and therapeutic heat application can also be employed to enhance muscle contraction and improve overall function [6,7,8,9].

As for surgical treatment, which mainly involves nerve surgery and plastic surgery, it is believed to be reserved for cases of PFP with a non-inflammatory etiology. The treatment of choice, when needed, is the injection of botulinum toxin into the orbicularis oculi muscle [1]. Although a significant percentage of cases resolve spontaneously within 2 to 3 weeks, the long-term prognosis is uncertain, which is why early treatment is recommended in all cases [10].

In Spain, the management of PFP begins with a careful assessment in the emergency department to exclude a central cause of the paralysis, and if no central etiology is identified, further evaluations are conducted to rule out any peripheral pathologies, primarily through the otolaryngology department [1]. This specialty is responsible for examining the middle ear, external auditory canal, auricular pavilion, and parotid region, where the facial nerve passes, to rule out any local pathology that could be causing the paralysis [2]. Once a diagnosis of either primary or secondary PFP is confirmed, patients are referred to rehabilitation services if the paralysis does not resolve spontaneously. Rehabilitation interventions are tailored to the individual needs of the patient, focusing on addressing specific deficits, restoring facial symmetry, and preventing long-term complications such as synkinesis [10].

Despite existing clinical guidelines, the management of PFP can vary widely across hospitals in Spain [1]. Large hospitals often have dedicated units for facial nerve disorders, with specialized personnel managing a high volume of cases. In contrast, smaller hospitals may lack such specialized units, making it essential to investigate how these institutions can improve their management protocols. One of the goals of this study is to explore the referral processes and rehabilitation pathways for patients with PFP, focusing on the timing of interventions and their content. This will allow us to propose a standardized approach to care, which could help optimize outcomes for patients in smaller hospitals. Moreover, understanding the reasons why patients often seek medical help from otolaryngologists rather than neurologists is crucial for improving the initial referral process and streamlining the patient’s journey through the healthcare system.

Understanding the sociodemographic and clinical profile of patients with PFP referred to rehabilitation services is crucial for optimizing treatment and prognosis. Factors such as age, gender, comorbidities, and the etiology of the paralysis significantly influence prognosis as well as treatment response [4]. Moreover, detailed knowledge of these characteristics enables the design of more personalized and effective intervention plans that address not only physical impairments but also the emotional and social implications of this condition, ultimately improving patients’ quality of life and reducing the impact of long-term sequelae [10,11].

Therefore, this study aims to describe the clinical and sociodemographic characteristics of patients with PFP treated in medical rehabilitation, as well as to evaluate the factors associated with the initial degree of impairment.

## 2. Materials and Methods

### 2.1. Study Design and Participants

A descriptive, cross-sectional study was conducted to analyze the sociodemographic, clinical, and functional characteristics of patients with PFP treated at the Rehabilitation Service of the University Hospital of Burgos (HUBU).

A convenience sampling method was used, including all patients diagnosed with PFP who attended medical rehabilitation from July 2018 to July 2023 after being referred from other medical services that identified the need for rehabilitative care during their respective clinical evaluations. All patients who agreed to participate in the study by signing the corresponding informed consent were included.

The research plan was submitted to the Medical Research Ethics Committee of the Burgos Health Area, and the required authorization was obtained with registration number CEIm 2576.

Data collection was carried out by the rehabilitation physician responsible for the study, who followed a previously established protocol and presented it to the Medical Research Ethics Committee. The data were anonymized to ensure patient confidentiality. The study complies with the ethical principles outlined in the Declaration of Helsinki. Data processing adhered to the European General Data Protection Regulation (GDPR) and Organic Law 3/2018 on Personal Data Protection and Guarantee of Digital Rights.

#### 2.1.1. Inclusion Criteria

- Patients diagnosed with PFP (either primary or secondary) who attended medical rehabilitation between July 2018 and July 2023.
- Patients with PFP treated at the Rehabilitation Service of the HUBU.
- Patients referred from other medical services that identified the need for rehabilitative care during their clinical evaluations (e.g., otolaryngology, neurology, etc.).
- Patients who voluntarily agreed to participate in the study by signing the informed consent form.

#### 2.1.2. Exclusion Criteria

- Patients with a prior history of PFP on the affected side.
- Patients with severe comorbidities, such as stroke or other neurological conditions that could impact the assessment of PFP.
- Patients who did not provide informed consent to participate in the study.
- Patients with conditions that could interfere with the rehabilitation process, such as severe cognitive impairments or psychiatric disorders that affect participation in the study.

### 2.2. Procedure

All patients referred to the rehabilitation service at HUBU during the study period who met the inclusion and exclusion criteria were included in the research, resulting in a total sample of 45 patients. The recruitment was notably influenced by the COVID-19 pandemic, which limited the arrival of new consultations and led to the inclusion of patients already under follow-up. Although modest, the sample size is consistent with previous studies in this area [12]. It also reflects real-life variability in both etiology and time to rehabilitation access.

Initial clinical and sociodemographic data were collected from the patients’ medical records and through the initial evaluation conducted during their first visit. The assessment of the initial degree of impairment was carried out using the Sunnybrook Facial Grading System (SFGS), a validated tool for evaluating facial function based on three components: static symmetry, voluntary movement, and the presence of synkinesis [13,14].

### 2.3. Instruments

Sociodemographic data, such as gender, age, and referring service, were collected during the initial patient interview and from their medical history. The main referring services were plastic surgery, primary care, otorhinolaryngology, emergency services, ophthalmology, neurology, and family medicine.

Regarding clinical variables, information was gathered on the affected side, the etiology of the paralysis, the timing of the session, and the time elapsed between the onset of the lesion and the first visit. PFP was classified as primary or idiopathic and secondary if an underlying cause was found in the complementary tests (otorhinolaryngological examination, Magnetic Resonance Imaging. Within the secondary cases, subgroups were made based on the specific diagnosis, allowing for further detailing of this category.

The degree of initial impairment was also assessed using the SFGS scale [13,14]. This is a widely used tool for evaluating facial function in patients with PFP, providing a systematic assessment of three aspects: symmetry rest, symmetry voluntary movements, and synkinesis [13].

The static symmetry subscale evaluates facial features (mouth, eyes, eyebrows) with scores ranging from 0 to 4, where higher values indicate better symmetry [13]. Symmetry voluntary movements are assessed in five regions (forehead, eyes, cheeks, mouth, and neck), with scores ranging from 0 to 5, resulting in a total percentage that reflects symmetry [13]. Synkinesis measures involuntary associated movements with scores ranging from 0 to 3, based on severity [13].

The total SFGS score ranges from 0 (complete dysfunction) to 100 (normal function), making it a scale sensitive to clinical changes and useful for monitoring recovery [13]. Studies have reported a Cronbach’s alpha coefficient between 0.85 and 0.95, demonstrating excellent internal consistency, as well as high interobserver reliability, making it a valid and reproducible tool [14]. Although it is more precise than global scales like the House–Brackmann scale, it requires training to ensure consistent evaluations and may be influenced by the evaluator’s subjectivity [15].

### 2.4. Statistical Analysis

Descriptive analyses of the sample characteristics were performed, presenting categorical variables as absolute frequencies and percentages and continuous variables as means and standard deviations (SD). The normality of the dataset was assessed using the Kolmogorov–Smirnov test. The association between etiology and referring service, as well as between etiology and waiting time, was analyzed using Pearson’s chi-square test to identify possible significant relationships that may determine whether certain types of etiology are associated with a specific referring service or influence the waiting time from the onset of the lesion to the first medical rehabilitation visit. To determine if there were significant differences in the degree of deterioration according to the SFGS scale based on clinical and sociodemographic variables such as sex, laterality, referring service, and etiology, ANOVA was applied. A *p*-value of less than 0.05 was considered statistically significant for all tests conducted.

Statistical analyses were performed using IBM SPSS Statistics version 28 (IBM Inc., Chicago, IL, USA). All analyses were conducted following rigorous statistical standards to ensure the validity of the results obtained.

## 3. Results

### 3.1. Sociodemographic and Clinical Characteristics of the Sample

The study was conducted with a total sample of 45 people with PFP, aged between 13 and 87 years (54.87 ± 19.897), with only one of them being a minor. Table 1 shows the frequencies and percentages of the categorical study variables. The percentage of patients was relatively homogeneous in terms of gender, with 17 women (37.28%) and 28 men (62.22%). The majority of the PFP cases were referred from the otorhinolaryngology department (64.44%), followed by the plastic surgery department (20%). Within the sample, there were services with minimal cases of PFP, such as the neurosurgery, ophthalmology, and pediatric neurology departments, each referring one patient, representing 2.22% in each case. The etiology was mostly idiopathic, accounting for 51.11% of the sample, although the remaining 20% of cases were of post-surgical etiology. More than half of the participants attended the rehabilitation service within the first two months following the lesion (60%), and nearly the entire sample attended before the sixth-month post-lesion (86.67%).

The mean score of the SFGS scale and its subscales, as well as the maximum and minimum values, are shown in Table 2.

### 3.2. Inferential Analyses

#### 3.2.1. Comparison of SFGS Between Groups

Inferential analyses were conducted to determine if there were significant differences in the degree of impairment according to the SFGS scale, as well as in its dimensions, based on categorical variables (sex, laterality, referring service, and etiology).

No statistical analysis was significant (*p* > 0.05). In other words, no statistically significant differences were found in the SFGS scale scores based on sex, laterality, referring service, and etiology.

#### 3.2.2. Association Between Etiology and Referring Service

Table 3 shows a statistically significant association between the etiology of PFP and the referring service, with a bilateral asymptotic significance of *p* = 0.033. The results reveal that, compared to expectations, fewer patients with post-surgical PFP and PFP due to abnormal growth of the parotid glands are referred from the otolaryngology service. In contrast, the analysis shows a higher number of patients with idiopathic PFP referred from the Primary Care service compared to what was expected.

Additionally, it is observed that, for both idiopathic and post-surgical PFP, the number of patients referred from the Plastic Surgery service is lower than expected. However, in the case of PFP due to abnormal growth of the parotid glands, this service refers more patients than anticipated. Finally, the analysis shows that the number of patients with post-surgical PFP referred from the Neurosurgery service exceeds the initial expectations.

#### 3.2.3. Association Between Etiology and Waiting Time from the Onset of the Lesion to the First Medical Rehabilitation Visit

Table 4 shows a significant association between the etiology of PFP and the time elapsed from the onset of the lesion to the first medical rehabilitation visit, with a significance of *p* = 0.028. In the cross-tabulation analysis, it is observed that patients with idiopathic PFP present a higher number of cases than expected in the 3-to-6-months category from the onset of the lesion to the medical rehabilitation visit. On the other hand, individuals with PFP caused by abnormal growth of the parotid glands exceed expectations in the category of up to 1 year of waiting time from the onset of symptoms to the first visit.

Additionally, cases of post-surgical PFP show a lower number of patients than expected who take between 13 and 24 months. Finally, subjects with PFP associated with an acoustic neuroma show a higher number of cases than expected in the category of more than 25 months from the onset of the lesion to the medical rehabilitation visit.

## 4. Discussion

This research provides valuable information on the sociodemographic and clinical characteristics of patients with PFP treated at the Rehabilitation Service of the HUBU.

The study results reveal a higher proportion of affected men (60.9%) compared to women (37%), consistent with existing literature, which reports variable outcomes depending on the study [16,17]. Regarding the average age, the findings also reflect a typical age range for this condition, particularly for idiopathic etiologies, which tend to manifest in middle-aged and older adults [17,18]. Conversely, cases in children and adolescents are more frequently associated with secondary PFP caused by infections or trauma [19].

Additionally, the predominance of idiopathic etiology aligns with the existing literature, which identifies the herpes simplex virus as the main cause of idiopathic PFP [3,5,10,20]. However, the results also highlight less frequent etiologies, such as PFP due to abnormal growth of the parotid glands or acoustic nerve neurinoma.

A more detailed analysis of gender distribution by etiology revealed that, among the 23 patients with idiopathic peripheral facial palsy, 14 were men (60.9%) and 9 were women (39.1%). In the secondary etiology group, which included 22 patients, 13 were men (59.1%) and 9 were women (40.9%). These results show a similar gender ratio across both etiological groups, suggesting that, in this sample, there are no substantial differences in sex distribution according to the cause of the palsy. However, further studies with larger sample sizes are recommended to confirm whether these proportions persist and to identify any significant associations between sex and the etiology of peripheral facial palsy.

Overall, the findings show a higher prevalence of cases referred from the otorhinolaryngology service (n = 29), followed by plastic surgery (n = 9) and primary care (n = 4). This study also identifies specific referral patterns based on the etiology of PFP. A lower number of patients with postsurgical paralysis and paralysis due to abnormal growth of the parotid glands were referred from the otorhinolaryngology service, despite having the highest number of overall referrals. Previous studies recognize otorhinolaryngology as one of the main specialties responsible for the initial management and referral of these patients, given their expertise in diagnosing and treating cranial nerve disorders, including PFP, which frequently has ontological origins [1,21,22]. However, these etiologies are often treated directly by specialties like neurosurgery or plastic surgery [8,10,23].

Regarding the plastic surgery service, the analysis revealed fewer referrals than expected for idiopathic and postsurgical cases but a higher than expected number for cases involving abnormal growth of the parotid glands. This pattern aligns with trends in other studies on this specialty, which often handles complex postsurgical etiologies or cases requiring reconstructive intervention [4,6,24,25].

On the other hand, idiopathic PFP cases showed a higher number of referrals from primary care than expected. The literature agrees that this etiology is the most referred from primary care services [3,26]. However, when analyzing general cases referred from primary care (n = 4), a lower rate was observed compared to other studies, potentially reflecting underutilization of this service or a lack of awareness about the importance of referring this condition to the rehabilitation service.

The waiting time between symptom onset and the first rehabilitation visit also revealed significant associations in inferential analyses. Typically, this period is less than two months [4,5,8,27]. However, results indicate that cases with complex etiologies, such as acoustic neurinoma, can exceed 25 months in some instances. This delay may stem from the more apparent clinical presentation of idiopathic etiologies, which professionals are more familiar with [10]. Moreover, the marked delay in rehabilitative care for acoustic neurinoma etiologies underscores well-documented diagnostic barriers for this condition, often presenting with symptoms that delay identification [28].

These findings highlight the importance of early identification of the causes of this condition to optimize treatment and prevent long-term consequences, as highlighted in existing literature, which recognizes preventive practices as critical for improving prognosis [4,8,9,29].

Additionally, the use of the SFGS scale as an evaluation tool provides a comprehensive measurement of facial function, assessing symmetry at rest, voluntary movements, and synkinesis [13,14]. However, inferential analyses to identify potential differences in functionality across various categorical variables (sex, age, referring service, laterality, and etiology) did not provide significant results. These findings conflict with existing literature, which identifies certain factors—such as female sex, hypertension, diabetes mellitus, postsurgical etiology, or previous paralysis—as predictors. Furthermore, studies reveal that PFP in children and young patients results in poorer outcomes regardless of etiology [27,29].

Early referral and the rapid initiation of therapeutic interventions are therefore crucial, as they can significantly impact the patient’s functional and emotional recovery, ultimately improving their quality of life. Previous studies indicate that facial asymmetry, difficulty in emotional expression, and impairments in speech and eating can lead to anxiety, depression, and reduced self-esteem [30,31]. Moreover, the social perception of the disease may contribute to isolation and decreased participation in work and recreational activities [32]. In this context, it would be beneficial for the treatment of PFP to also incorporate psychological and social support strategies, aiming to enhance the patient’s adaptation to their condition and promote better overall outcomes.

Although the present study presents several strengths, such as addressing an un-derstudied topic and offering a detailed analysis of the referral pathways and access times to rehabilitation services for patients with PFP, a few limitations must also be acknowledged.

First, the available sample size and the Eurocentric, single-center nature of the study limit the generalizability of the findings. The heterogeneity inherent to this pathology—regarding epidemiology, etiology, and treatment approaches—further restricts the ability to draw broader conclusions. Additionally, the study population reflects the characteristics of patients attending the rehabilitation service at the Hospital of Burgos, and although the results may be extrapolated to similar hospitals, they should be interpreted with caution when applied to other contexts with different healthcare structures or patient profiles.

Moreover, the under-representation of patients referred directly from primary care highlights a possible gap in the early detection and referral process, suggesting the need for improved medical training and structured strategies to facilitate early access to re-habilitation services. Finally, given the dynamic nature of facial paralysis and its variable progression over time, future research should consider longitudinal designs with larger, multicenter cohorts to better explore the evolution and recovery trajectories in diverse clinical settings.

## 5. Conclusions

This research identifies idiopathic etiology as the most common among patients with PFP treated in rehabilitation, with a higher prevalence in men and older adults, while infectious or traumatic causes predominate in children. Otorhinolaryngology stands out as the primary referring service, although underutilization of primary care for case referrals is noted. Furthermore, prolonged times to initiate rehabilitation in complex etiologies reflect diagnostic barriers, highlighting the importance of early identification. Lastly, the need for future studies with larger sample sizes is emphasized to adequately evaluate factors associated with the initial degree of impairment and optimize the clinical management of this condition.

## Figures and Tables

**Table 1:** Sociodemographic and clinical characteristics of the sample.

Sociodemographic and Clinical Characteristics		N (%)
Gender	Male	28 (62.22%)
	Female	17 (37.78%)
Referring Service	Otorhinolaryngology	29 (64.44%)
	Primary Care	4 (8.89%)
	Plastic Surgery	9 (20.00%)
	Neurosurgery	1 (2.22%)
	Ophthalmology	1 (2.22%)
	Pediatric Neurology	1 (2.22%)
Etiology	Herpes Zoster Virus	5 (11.11%)
	Pregnancy	1 (2.22%)
	Idiopathic	23 (51.11%)
	Post-surgical	9 (20.00%)
	COVID-19	2 (4.44%)
	Acoustic neuroma	3 (6.67%)
	Abnormal growth of the parotid gland	2 (4.44%)
Laterality	Left	23 (51.11%)
	Right	22 (48.89%)
Waiting time from the onset of the lesion to the first medical rehabilitation visit	≤2 months	27 (60.00%)
	3–6 months	12 (26.67%)
	7–12 months	1 (2.22%)
	13–24 months	2 (4.44%)
	25 months	3 (6.67%)

**Table 2:** SFGS score.

Category	Mean	Maximum	Minimum
SFGS Resting Symmetry	9.89	20	0
SFGS Symmetry of Voluntary Movement	57.16	96	0
SFGS Synkinesis	3.86	7	0
SFGS Total	46.20	92	6

SFGS: Sunnybrook Facial Grading System.

**Table 3:** Chi-square test to determine the association between sending service and etiology.

		ENT	PC	PS	NS	OPTH	PN
**Herpes Zoster Virus**	Count	5	0	0	0	0	0
	Expected count	3.2	0.4	1.0	0.1	0.1	0.1
	Corrected residual	1.8	−0.7	−1.2	−0.4	−0.4	−0.4
**Pregnancy**	Count	1	0	0	0	0	0
	Expected count	0.6	0.1	0.2	0	0	0
	Corrected residual	0.8	−0.3	−0.5	−0.2	−0.2	−0.2
**Idiopathic**	Count	17	4	0	0	1	1
	Expected count	14.8	2	4.6	0.5	0.5	0.5
	Corrected residual	1.4	2.0	−3.4	−1.0	1.0	1.0
**Post-surgical**	Count	1	0	7	1	0	0
	Expected count	5.8	0.8	1.8	0.2	0.2	0.2
	Corrected residual	−3.7	−1.0	4.8	2.0	−0.5	−0.5
**COVID-19**	Count	2	0	0	0	0	0
	Expected count	1.3	0.2	0.4	0	0	0
	Corrected residual	1.1	−0.5	−0.7	−0.2	−0.2	−0.2
**Acoustic Neuroma**	Count	3	0	0	0	0	0
	Expected count	1.9	0.3	0.6	0.1	0.1	0.1
	Corrected residual	1.3	−0.6	−0.9	−0.3	−0.3	−0.3
**Abnormal growth of the parotid gland**	Count	0	0	2	0	0	0
	Expected count	1.3	0.2	0.4	0	0	0
	Corrected residual	−1.9	−0.5	2.9	−0.2	−0.2	−0.2

ENT: Otorhinolaryngology; PC: Primary Care; PS: Plastic Surgery; NS: Neurosurgery; OPTH: Ophthalmology; PN: Pediatric Neurology. X^2^ (30,45) = 45,700, *p* = 0.033.

**Table 4:** Chi-square test to determine the association between etiology and waiting time from the onset of the lesion to the first medical rehabilitation visit.

		≤2 Months	3–6 Months	7–12 Months	13–24 Months	>25 Months
**Herpes Zoster Virus**	Count	4	1	0	0	0
	Expected count	3.0	1.3	0.1	0.2	0.3
	Corrected residual	1.0	−0.4	−0.4	−0.5	−0.6
**Pregnancy**	Count	1	0	0	0	0
	Expected count	0.6	0.3	0	0	0.1
	Corrected residual	0.8	−0.6	−0.2	−0.2	−1.3
**Idiopathic**	Count	13	9	0	0	1
	Expected count	13.8	6.1	0.5	1.0	1.5
	Corrected residual	−0.5	1.9	−1.0	−1.5	−0.6
**Post-surgical**	Count	5	1	0	2	1
	Expected count	5.4	2.4	0.2	0.4	0.6
	Corrected residual	−0.3	−1.2	−0.5	20.9	−0.6
**COVID-19**	Count	1	1	0	0	0
	Expected count	1.2	0.5	0	0.1	0.1
	Corrected residual	−0.3	0.8	−0.2	−0.3	−0.4
**Acoustic** **Neuroma**	Count	2	0	0	0	1
	Expected count	1.8	0.8	0.1	0.1	0.2
	Corrected residual	0.2	−1.1	−0.3	−0.4	1.9
**Abnormal growth of the parotid gland**	Count	1	0	1	0	0
	Expected count	1.2	0.5	0	0.1	0.1
	Corrected residual	−0.3	−0.9	4.7	−0.3	−0.4

X^2^ (24,45) = 38,832, *p* = 0.028.

## Data Availability

Due to privacy and ethical restrictions, the data are not publicly available.
